# Comprehensive analysis of expression, prognosis and immune infiltration for TIMPs in glioblastoma

**DOI:** 10.1186/s12883-021-02477-1

**Published:** 2021-11-15

**Authors:** Jinkun Han, Yajun Jing, Fubing Han, Peng Sun

**Affiliations:** Department of Neurosurgery, the Affiliated Hospital of Qingdao University, Qingdao, China

**Keywords:** Bioinformatics analysis, Tissue inhibitors of metalloproteinases family, Glioblastoma, Biomarker, Prognosis, Immune infiltration

## Abstract

**Background:**

Tissue inhibitors of metalloproteinase (TIMP) family proteins are peptidases involved in extracellular matrix (ECM) degradation. Various diseases are related to TIMPs, and the primary reason is that TIMPs can indirectly regulate remodelling of the ECM and cell signalling by regulating matrix metalloproteinase (MMP) activity. However, the link between TIMPs and glioblastoma (GBM) is unclear.

**Objective:**

This study aimed to explore the role of TIMP expression and immune infiltration in GBM.

**Methods:**

Oncomine, GEPIA, OSgbm, LinkedOmics, STRING, GeneMANIA, Enrichr, and TIMER were used to conduct differential expression, prognosis, and immune infiltration analyses of TIMPs in GBM.

**Results:**

All members of the TIMP family had significantly higher expression levels in GBM. High TIMP3 expression correlated with better overall survival (OS) and disease-specific survival (DSS) in GBM patients. TIMP4 was associated with a long OS in GBM patients. We found a positive relationship between TIMP3 and TIMP4, identifying gene sets with similar or opposite expression directions to those in GBM patients. TIMPs and associated genes are mainly associated with extracellular matrix organization and involve proteoglycan pathways in cancer. The expression levels of TIMPs in GBM correlate with the infiltration of various immune cells, including CD4+ T cells, macrophages, neutrophils, B cells, CD8^+^ T cells, and dendritic cells.

**Conclusions:**

Our study inspires new ideas for the role of TIMPs in GBM and provides new directions for multiple treatment modalities, including immunotherapy, in GBM.

**Supplementary Information:**

The online version contains supplementary material available at 10.1186/s12883-021-02477-1.

## Introduction

Glioblastoma (GBM) is the most aggressive of the four grades of gliomas classified by WHO [1] and accounts for 17% of all primary brain tumours [2]. When radiotherapy was combined with temozolomide, the survival time of GBM patients reached 14.6 months, and the 2-year OS and 5-year OS rates were 27.2 and 9.8%, respectively [3]. Matrix metalloproteinases (MMPs) are a family of zinc-dependent endopeptidases implicated in degrading components of the extracellular matrix (ECM) [4], tumour cell invasion and angiogenesis, and suppression of antitumour immune surveillance [5]. Previous studies have found that MMPs are associated with different tumour types, such as papillary thyroid carcinomas [6] and lung adenocarcinoma [7]. Several studies have indicated that MMPs are related to increased invasiveness [8] and a poor prognosis [9] in GBM. Tissue inhibitors of metalloproteinases (TIMPs) comprise four natural endogenous secreted proteins that inhibit the activity of MMPs [10]. Therefore, TIMPs regulate cellular processes such as migration, proliferation, and survival by controlling ECM degradation via interaction with MMPs [11]. In addition to this originally described feature [12], over the past decade, many additional relatively independent biological activities have been identified, including cell growth [13], apoptosis [14], differentiation [15], angiogenesis [16], and proliferation [17].

The TIMP gene family has been studied in various tumours, including GBM, but no systematic analysis has investigated the relationship between their expression and prognosis in glioblastoma. With the development of microarray technology, immune infiltration research, and the progress of single-cell technology, using bioinformatics analysis is vital to examine the expression pattern and prognostic value of the TIMP gene family in glioblastoma and to use cutting-edge technology and related research data for further analysis. This process will also lead to an update of our understanding of this ancient family.

## Materials and methods

### Oncomine

Oncomine (https://www.oncomine.org/resource/login.html) is a platform with powerful analysis functions that compute gene expression signatures, clusters, and gene-set modules from the data [18]. We analysed the mRNA expression of TIMPs and compared the difference between the normal and tumour groups by Student’s *t*-test in various tumours on this platform. A *p* value < 0.05 and fold change ≥2 for the p value indicated that the difference between the normal and tumour groups was statistically significant.

### GEPIA2

GEPIA2 (http://gepia.cancer-pku.cn/index.html) is an updated version of Gene Expression Profiling Interactive Analysis (GEPIA) that processes mRNA data from The Cancer Genome Atlas (TCGA) and Genotype-Tissue Expression (GTEx) projects [19]. The website has various customized functions, such as similar genetic testing, correlation analysis, and dimensionality reduction analysis. In our study, we performed differential expression analysis of family genes as a validation set.

### OSgbm

OSgbm (http://bioinfo.henu.edu.cn/GBM/GBMList.jsp.) is a web server that performs online survival analysis of glioblastoma [20]. This website comprises 684 samples with transcriptome profiles and clinical information from TCGA, Gene Expression Omnibus (GEO), and Chinese Glioma Genome Atlas (CGGA). We used it to explore the impact of TIMPs on the prognosis of GBM patients. The survival analysis results are presented as Kaplan–Meier (KM) plots with hazard ratios (HRs) and log-rank plots with *p* values.

### LinkedOmics

LinkedOmics (http://www.linkedomics.orglogin.php) is a public portal for multiomics data that mainly includes TCGA cancer types [21]. The mutual combination of different modules allows the analysis of multiomics data from various cancers. Here, we explored the expression relationships between TIMPs and different genes in GBM.

### Gliovis

Gliovis (http://gliovis.bioinfo.cnio.es/) is a user-friendly website tool that includes brain tumour research results containing more than 6,500 tumour samples, approximately 50 expression datasets, and mainly gliomas [22]. We explored GBM data from the TCGA database at this server to investigate the correlation between the expression of TIMP family gene members and GBM subgroups according to different classifications.

### STRING

STRING (https://string-db.org/) is a website to perform protein interactions. Its goal is to build a comprehensive, objective global network that can perform direct (physical) and indirect (functional) interactions of proteins. We used it to generate protein–protein interaction (PPI) networks of TIMPs.

### GeneMANIA

GeneMANIA (https://genemania.org/) is a server that performs gene association and similar gene exploration [23]. It can extend the submitted gene list by analysing a large amount of association data in the background and the gene association function in the list. We found the associated genes of TIMPs here and explored their different functions with GeneMANIA.

### Enrichr

Enrichr (https://maayanlab.cloud/Enrichr/) is a search engine that performs multiple functional enrichment analyses of input genes by querying many collections of annotated genes [24–26]. We performed functional analysis here for the set of genes generated in GeneMANIA.

### cBioPortal

cBioPortal (http://cbioportal.org) is an open portal to explore and analyse multidimensional data from different cancers and combine molecular data and clinical data with visualization. We used this database to explore the mutation of TIMP family genes in GBM and link them to prognosis.

### TIMER

TIMER (http://timer.cistrome.org/) is a user-friendly portal that evaluates the molecular characterization of tumour interactions with immunity [27]. It calculates the level of immunity in various infiltrating tumours in advance and provides six main analysis modules so that the relationship between tumours and immunity can be interactively explored. We conducted a correlative analysis of TIMPs at this site for measures of immune infiltration, including tumour purity and six immune cells in GBM.

## Results

### Transcriptional levels of TIMPs in patients with GBM

**Fig. 1:**
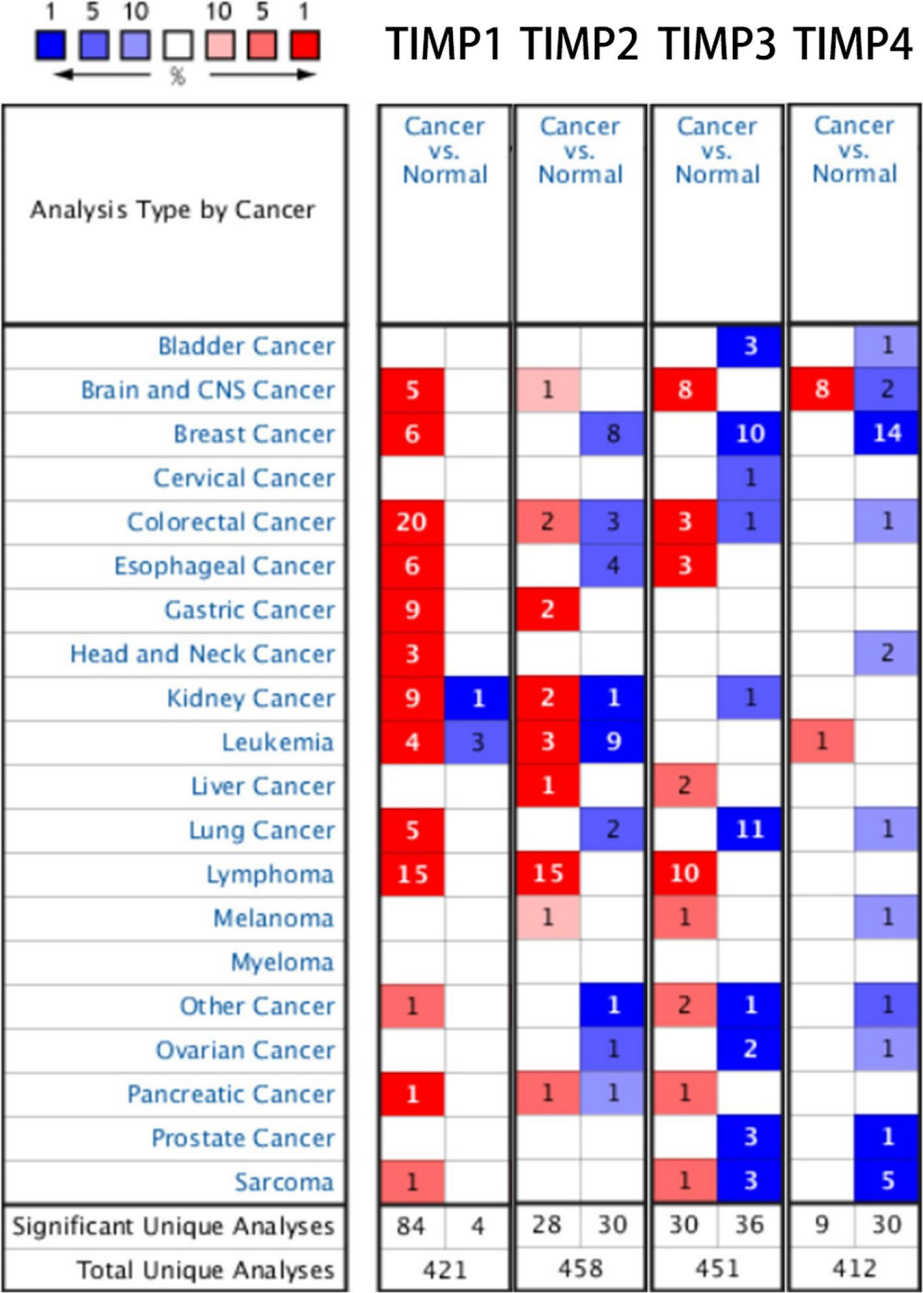
mRNA expression of TIMP family members in different cancers

Four TIMP factors have been identified in humans. We compared the transcriptional levels of TIMPs in GBM with those in normal samples using the filter of differential analysis in Glioblastoma vs. Normal and Brain Glioblastoma vs. Normal in ONCOMINE databases (Fig. 1). The mRNA expression levels of TIMP1 were significantly upregulated in patients with GBM in five datasets (Table 1). In detail, the expression level of TIMP1 in the tumour group was dramatically augmented with a fold change of 18.922 in Bredel Brain 2’s dataset [28]. Additionally, the lowest fold change of 2.419 was found in the differential comparison between GBM and normal samples of neural stem cells in Lee Brain’s dataset [29]. Regarding TIMP2, increased expression occurred in both TCGA Brain and Liang Brain [30] datasets (Table 1). The samples of these two datasets were brain vs. brain GBM, brain and cerebellum vs. GBM. The fold change of TIMP2 in TCGA Brain was 2.218, and the latter was 2.304. In Bredel Brain 2’s [28] and Lee Brain’s datasets [29], TIMP3 was overexpressed with a fold change≥2 (Table 1). TIMP4 was overexpressed in two types of samples in the TCGA Brain’s dataset: brain GBM vs. brain and GBM vs. brain. Their fold changes, 8.998 and 8.887, respectively, were both higher (Table 1). The lowest fold change of 2.774 appeared in Shai Brain’s dataset [31], and the sample of this dataset was GBM vs. White Matter.

**Table 1:** Significant changes in TIMP expression at the transcription level between different types of GBM and normal tissues (oncomine)

Gene ID	*p* Value 1	Fold change	*p* Value 2	t test	Sample	References
TIMP-1	8.87E-7	6.899	6.76E-14	12.843	GBM vs. Brain and Cerebellum	Liang Brain
		18.922	9.53E-13	12.271	GBM vs. Brain	Bredel Brain 2
		4.55	1.06E-7	6.588	GBM vs. White Matter	Shai Brain
		2.419	1.65E-6	6.096	GBM vs. Neural Stem Cell	Lee Brain
		5.188	1.24E-11	8.438	GBM vs. Brain	Sun Brain
TIMP2	0.006	2.218	6.09E-9	14.400	Brain GBM vs. Brain	TCGA Brain
		2.304	0.012	4.418	GBM vs. Brain and Cerebellum	Liang Brain
TIMP3	1.16E-5	9.042	1.48E-10	13.731	GBM vs. Neural Stem Cell	Lee Brain
		2.008	4.39E-8	8.106	GBM vs. Brain	Bredel Brain 2
TIMP4	2.33E-4	4.168	1.93E-13	12.220	GBM vs. Brain	Murat Brain [32]
		7.897	3.86E-19	11.720	GBM vs. Brain	Sun Brain [33]
		8.998	4.50E-4	6.316	GBM vs. Brain	TCGA Brain
		8.887	4.95E-9	14.307	Brain GBM vs. Brain	TCGA Brain
		2.774	1.51E-5	4.948	GBM vs. White Matter	Shai Brain

*p* Value 1: *p* Value in comparison across gene analyses

*p* Value 2: *p* Value in a gene analysis

### Differential expression of TIMPs in GBM

**Fig. 2:**
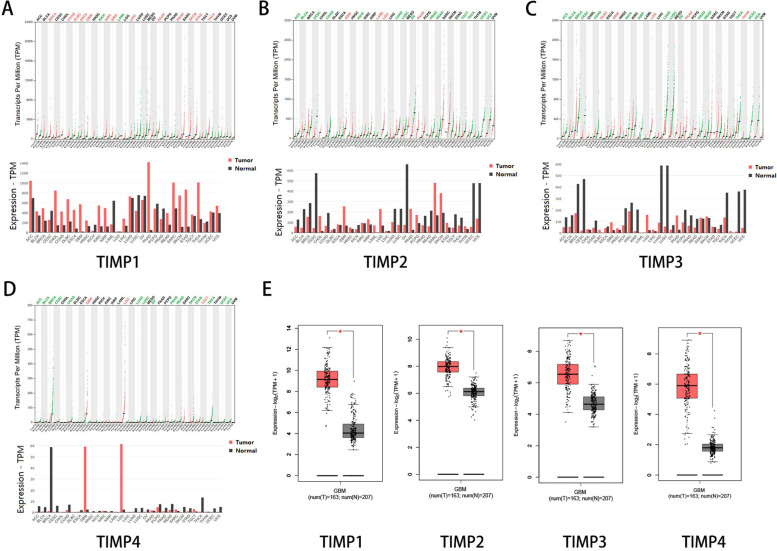
Expression of TIMPs in GEPIA2. TIMP1 expression in pan-cancer (**A**). TIMP2 expression in pan-cancer (**B**). TIMP3 expression in pan-cancer (**C**). TIMP4 expression in pan-cancer (**D**). Differential expression of TIMPs between GBM and normal samples (**E**). *: *p*<0.05

In the GEPIA (Gene Expression Profiling Interactive Analysis) dataset (http://gepia.cancer-pku.cn/), we compared the mRNA expression level of TIMPs between GBM and normal samples. The gene expression levels of TIMP1, TIMP2, TIMP3, and TIMP4 were all higher in GBM than in normal samples (Fig. 2A–E).

### The prognostic values of TIMPs in glioblastoma and co-expression genes of significant gene

**Fig. 3:**
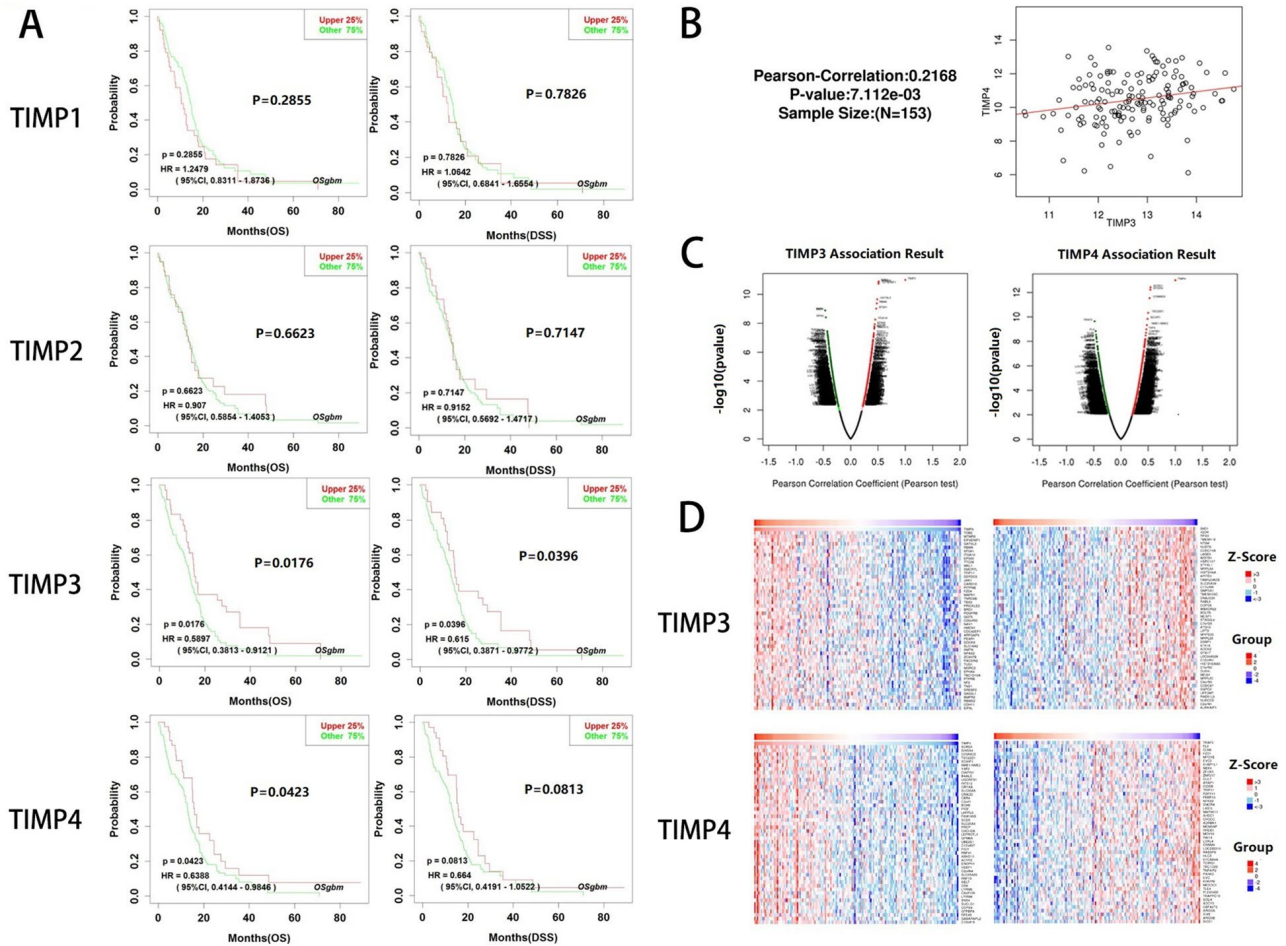
Prognostic value and related genes of TIMPs in GBM. Prognostic value of TIMPs in GBM (**A**). Correlation of TIMP3 and TIMP4 (**B**). Volcano plot of genes implicated in the expression of TIMP3 and TIMP4 in GBM (**C**). Heatmap of the top 50 genes positively and negatively correlated with the expression of TIMP3 and TIMP4 in GBM (**D**)

We investigated the prognostic value of TIMP1, TIMP2, TIMP3 and TIMP4 in GBM data from TCGA using OSgbm [20]. The TIMP3 overexpression group was associated with better overall survival (OS) and disease-specific survival (DSS) (Fig. 3A). TIMP4 overexpression was associated with better OS but without significance in DSS (Fig. 3A). Neither TIMP1 nor TIMP2 showed differences in OS and DSS (Fig. 3A). Next, we found a positive correlation between TIMP3 and TIMP4 expression in the LinkedOmics dataset (*R* = 0.2168, *p* <0.01) (Fig. 3B) [21].

Given the prognostic value of TIMP3 and TIMP4, using the functional module of LinkedOmics, we explored their associated genes in the TCGA_GBM cohort. The genes positively (dark red dots) and negatively (dark green dots) correlated with TIMP3 and TIMP4 are shown in Fig. 3C. The top 50 significant genes positively and negatively correlated with TIMP3 and TIMP4 are shown in the heat map (Fig. 3D). TIMP3 expression showed a positive association with the expression of TOB2 (positive rank #1; *r* = 0.513; *p* = 1.275e-11), MTMR3 (*r* = 0.511; *p* = 1.448e-11), and EIF4ENIF1 (*r* = 0.510; *p* = 1.832e-11). The top three genes with a positive correlation with TIMP4 were SCRG1 (*r* = 0.544; *p* = 3.778e-13), SHISA4 (*r* = 0.540; *p* = 5.826e-13) and COMMD2 (*r* = 0.526; *p* = 2.890e-12). These genes are more likely to be protective genes similar to TIMP3 and TIMP4.

### Validation of the expression of TIMPs in different subgroups of GBM

**Fig. 4:**
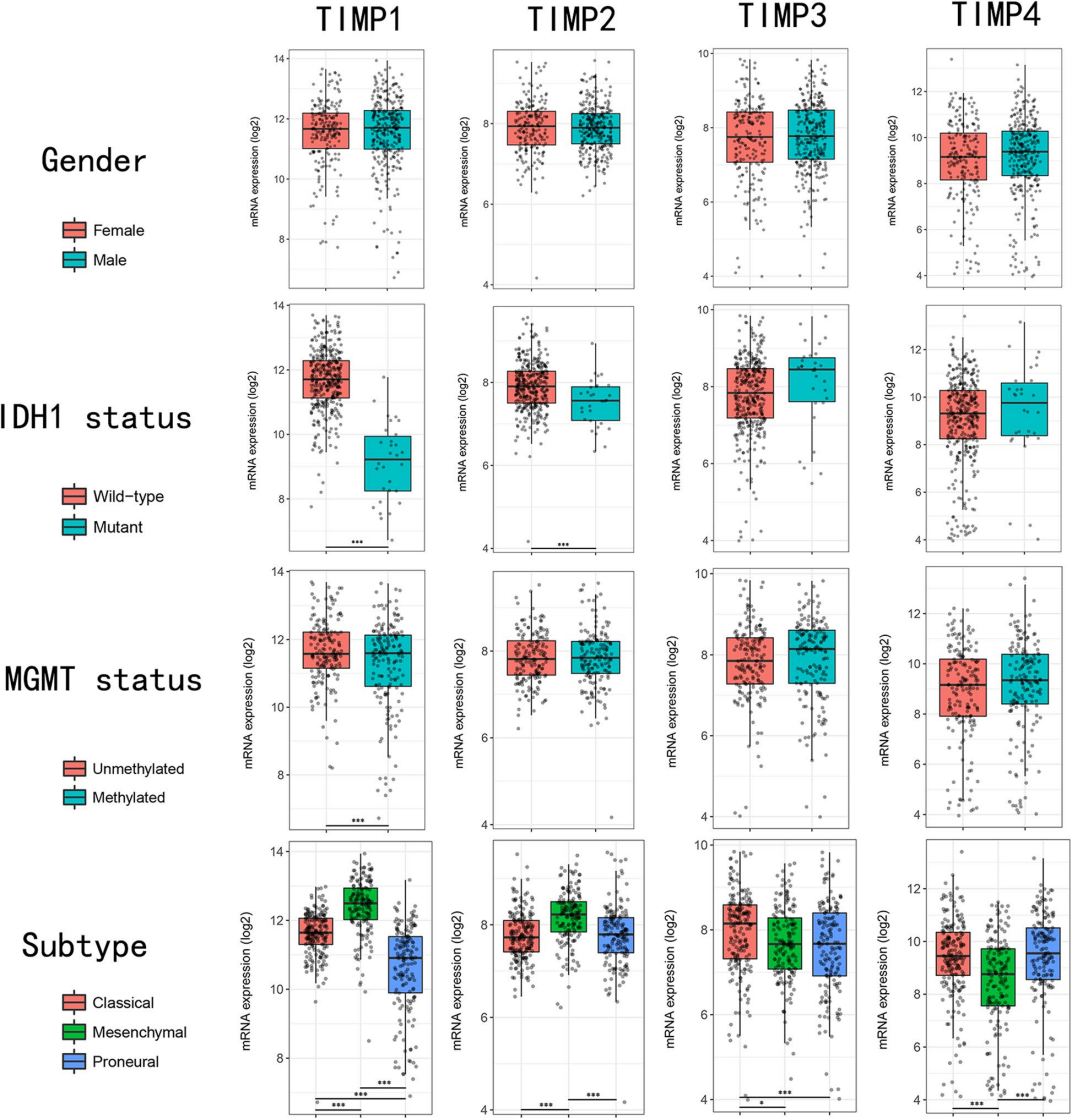
Correlations of TIMPS with subgroups. The expression data of TIMPs in GBM from TCGA were grouped according to sex, IDH1 status, MGMT status, and subtype. The correlation was verified using T-test. ***p<0.001; **p<0.01; *p<0.05

We divided the GBM data from TCGA into different subgroups according to different criteria in Gliovis. Next, we investigated whether TIMPs were differentially expressed between different subgroups (Fig. 4). The four TIMP family members were not differentially expressed between sexes.

The expression levels of both TIMP1 and TIMP2 were significantly decreased in patients with IDH1 mutations. However, no association was found between TIMP3 and TIMP4 expression and IDH1 mutation. Among the TIMP family, only TIMP1 expression correlated with methylation of the MGMT promoter. We focused on evaluating the differential expression of TIMPs between different subtypes of GBM. The four genes showed significant expression differences among classical, mesenchymal and proneural GBM. TIMP1 and TIMP2 expression was highest in the mesenchymal region, but TIMP3 and TIMP4 expression lower in this region. Specific data can be viewed in the Supplementary file.

### Protein-protein interaction analyses and functional enrichment analysis of TIMPs

**Fig. 5:**
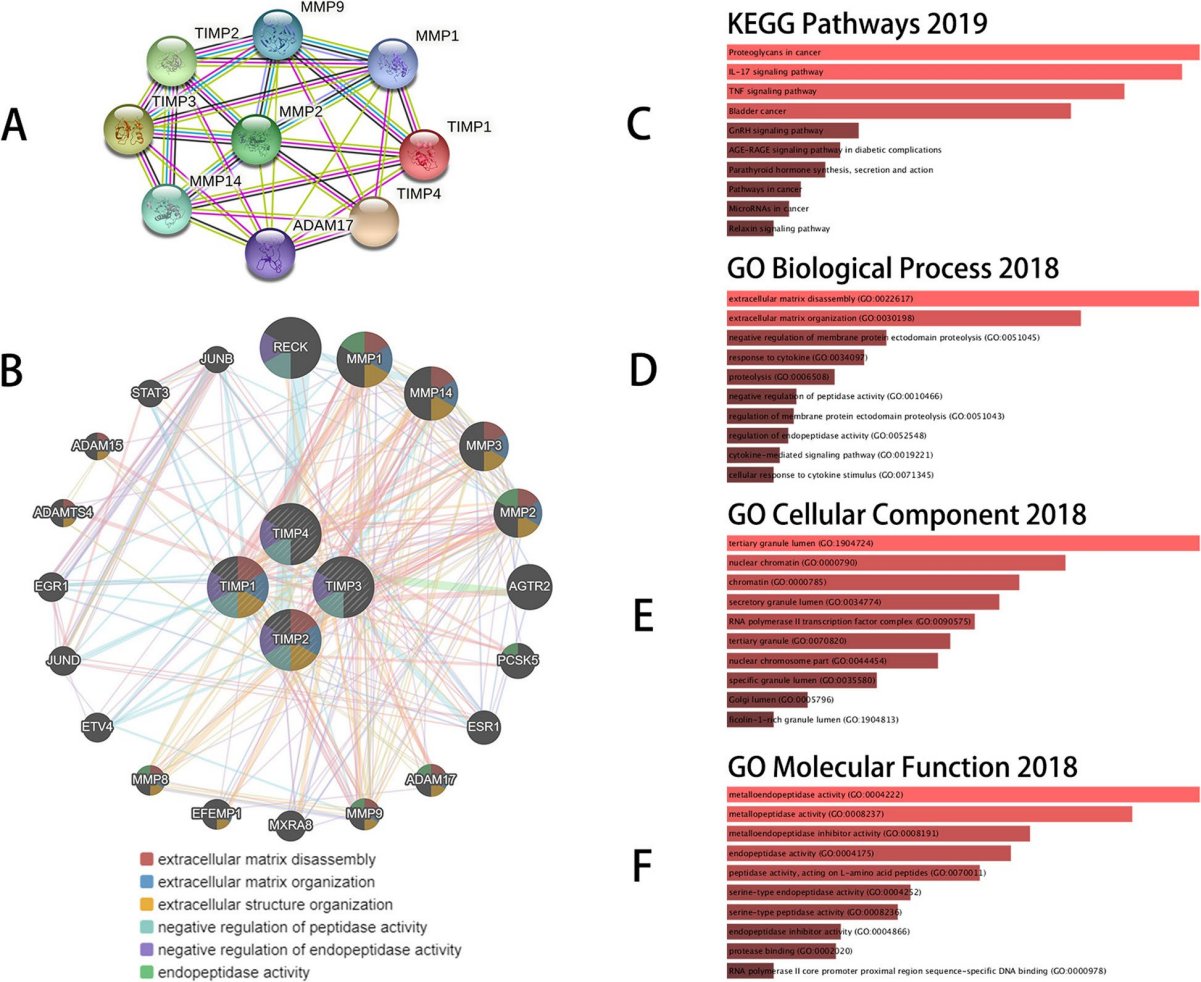
Interaction analysis and functional enrichment analysis of TIMPs. PPI network of TIMPs including 9 nodes and 29 edges (STRING) (**A**). Gene–gene interaction network of TIMPs (GeneMANIA) (**B**). Functional enrichment analysis of TIMPs and related genes in patients with GBM (**C**-**F**)

We performed protein–protein interaction PPI network analysis of TIMPs using STRING to explore the potential interactions among them. Eventually, we obtained a protein interaction network comprising 9 nodes and 29 edges (Fig. 5A). We also used the GeneMANIA database to identify the genes associated with TIMPs, revealing that the functions of TIMPs with 20 related molecules (such as RECK, MMP1, MMP14, MMP3, MMP2, AGTR2, and PCSK5) were primarily associated with extracellular matrix organization, extracellular structure organization, negative regulation of peptidase activity and endopeptidase activity (Fig. 5B). To further analyze the functions of TIMPs and related genes as well as to make a comparative control with the functions presented on GeneMANIA, we performed functional enrichment analysis of these genes at the Enrichr website. The results showed that the top 10 Kyoto Encyclopedia of Genes and Genomes (KEGG) pathways for TIMPs and their 20 correlated genes were mainly associated with proteoglycans in cancer, the IL-17 signalling pathway, the TNF signalling pathway, the GnRH signalling pathway, and others (Fig. 5C) [34]. These pathways may involve mechanisms of progression in GBM. Next, using the Enrichr tool, GO analysis was performed using TIMPs and their correlated genes to analyse functions in biological processes, molecular functions, and cellular components. TIMPs and correlated genes were mainly associated with extracellular matrix disassembly in biological processes (Fig. 5D), tertiary granule lumen in cellular components (Fig. 5E), and metalloendopeptidase activity in molecular functions (Fig. 5F).

### Genetic alteration of TIMPs in patients with GBM

**Fig. 6:**
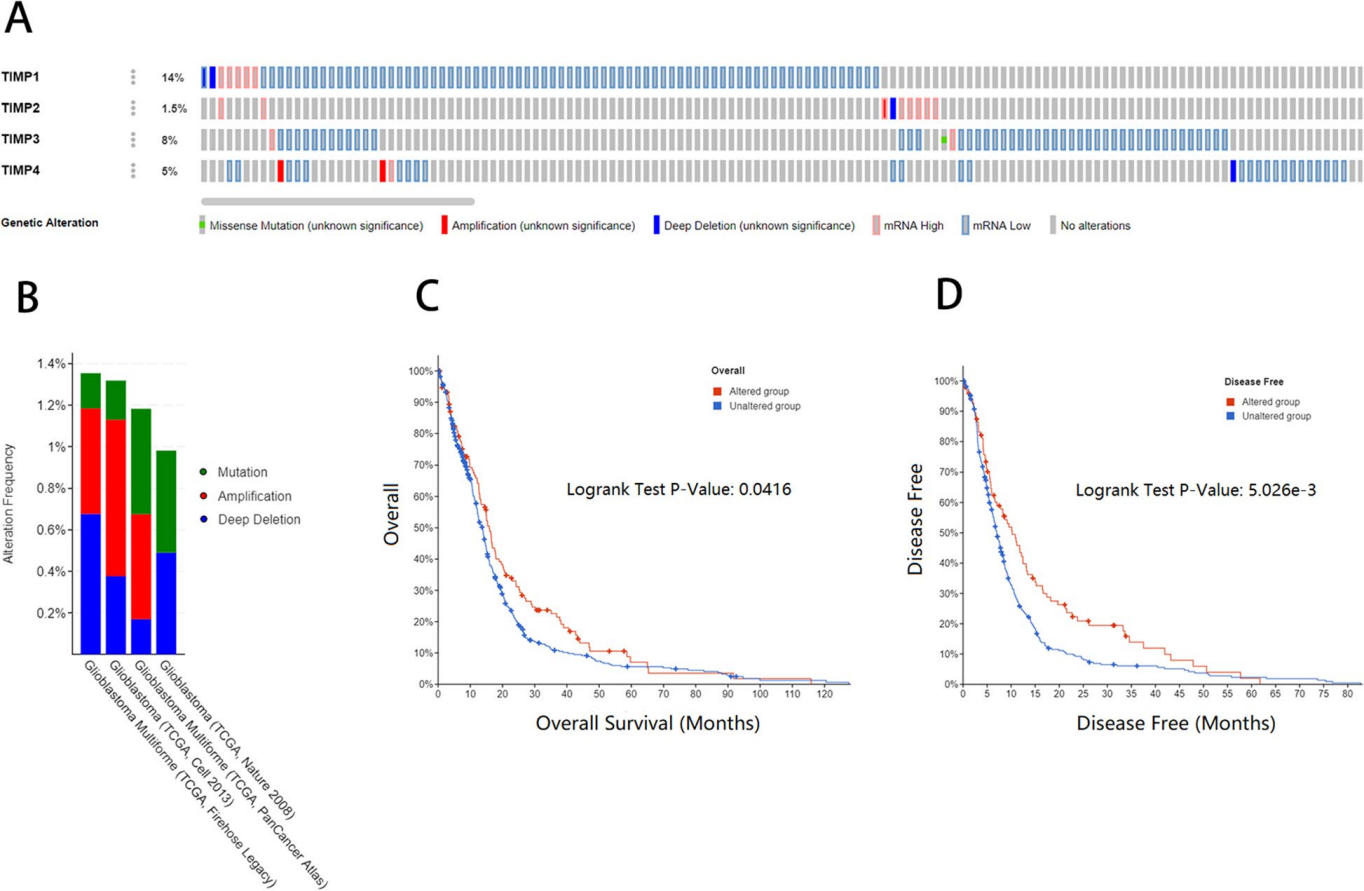
Genetic alterations linked to hub genes in THCA. Alteration frequencies of TIMPs (**A**). Genetic mutations frequencies of TIMPs in GBM (**B**). KM plots comparing OS in patients with and without TIMP alterations (**C**). KM plots comparing DFS in patients with and without TIMP alterations (**D**)

We analysed the genetic changes of TIMPs in the database. The TIMP gene was genetically altered in 23% of cases. The alteration frequency was 14% for TIMP1, 1.5% for TIMP2, 8% for TIMP3 and 5% for TIMP4 (Fig. 6A). However, these data primarily represent expression changes. We explored the mutation status of TIMPs in the four databases Glioblastoma (TCGA, Nature 2008), Glioblastoma Multiforme (TCGA, Firehose Legacy), Glioblastoma Multiforme (TCGA, PanCancer Atlas) and Glioblastoma (TCGA, Cell 2013) and found that the highest mutation frequency did not exceed 1.4% (Fig. 6B). We also performed correlation analysis between patients with genetic alterations in TIMPs and prognostic conditions in the database and found that the patients were significantly associated with better OS and DFS (Fig. 6C, D).

### Relationship between TIMPs expression and immune infiltration in GBM

**Fig. 7:**
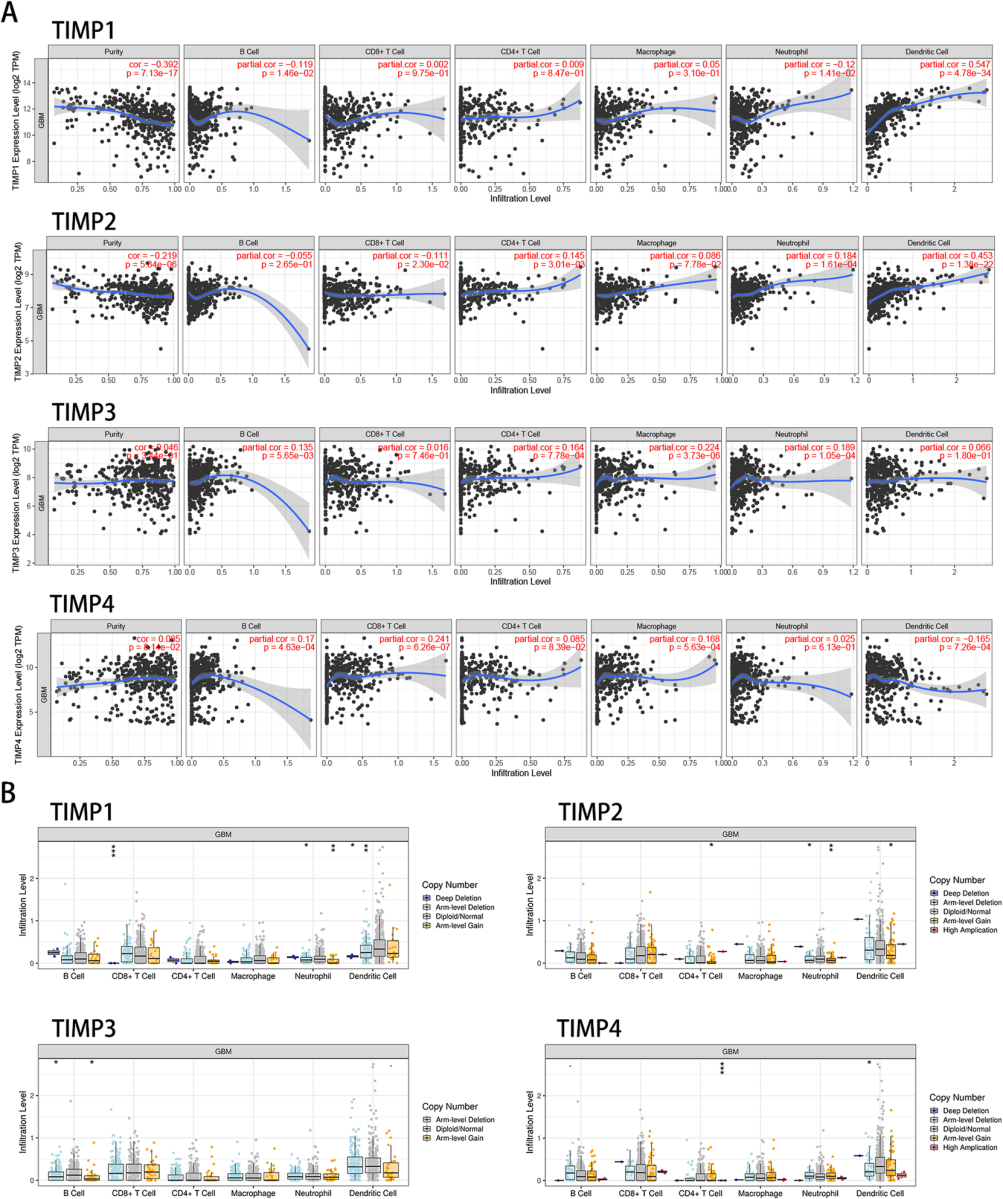
Association of the genetic expression of TIMPs with immune infiltration in GBM. Correlation between the abundance of immune cells and expression of TIMPs (**A**). Effect of the CNV of TIMPs on the distribution of various immune cells (**B**). (*p < 0.05; **p < 0.01; ***p < 0.001)

Immune infiltration is a component of the tumour microenvironment and is highly correlated with the diagnosis, progression, and prognosis of tumours. We used the TIMER database in this study to reveal the correlation between the gene expression levels of TIMP family members and immune cell infiltration in patients with GBM (Fig. 7A). TIMP1 expression was negatively associated with tumour purity but positively correlated with dendritic cells. Additionally, this correlation was also observed for TIMP2. TIMP3 expression was associated with B cells, CD4+ T cells, macrophages, and neutrophils. However, no negative results are shown for TIMP3. Finally, TIMP4 expression was positively associated with the infiltration of B cells, CD8+ T cells, and macrophages in GBM patients. We also explored the relationship between the copy number variation (CNV) of TIMPs and immune infiltration level (Fig. 7B). The association of CNV with immune infiltration varied among the four genes. The levels of CNS in TIMP1 and TIMP2 were associated with more immune infiltration indicators, and , their arm-level deletion and arm-level gain were closely related to neutrophils. Regarding the remaining two genes, TIMP3 and TIMP4, that affected the prognosis, the CNV level of TIMP3 was the least significantly associated with immune infiltration indicators but was the only member of the family whose CNV affected the immune infiltration level of B cells. The CNV of TIMP4 was significantly correlated with both CD4+ T cells and dendritic cells, a finding similar to that of TIMP2. However, the CNV showed no correlation with neutrophils.

## Discussion

Previous studies have found that TIMP gene family members are differentially expressed in multiple cancers [35–37]. Because MMPs are highly correlated with the prognosis of multiple cancers, TIMPs, at least theoretically, may affect prognosis in some cancer cases as specific inhibitors. In brain tumours, including glioma, many reports have investigated the differential expression of TIMPs [38–40]. However, previous reports on TIMPs in GBM are largely fragmented, and systematic analysis of the TIMP family in GBM is rare. In the present study, we performed systemic analysis concerning TIMPs for the prognosis of patients with GBM.

As shown in the results (Fig. 1), TIMPs were differentially expressed in brain and CNS cancer, colorectal cancer, and breast cancer, a finding that is also consistent with previous reports [35, 41]. Interestingly, the expression levels of these genes were almost all upregulated in brain and CNS cancer, but TIMP expression was downregulated in normal individuals (Fig. 2). TIMP-1 demonstrated both antitumour and antimetastatic functions in multiple genetic models [42, 43]. However, in our study, TIMP1 expression was not associated with the prognosis of GBM (Fig. 3A). Most of the studies on TIMP1 gene expression levels showed a negative relationship with prognosis [44, 45]. High TIMP-1 expression is strongly associated with a poor prognosis in almost all known cancer types [46]. Many explanations exist for this contradictory phenomenon, such as the interaction between TIMP1 and CD63 to activate MAPK signalling and participation in the HGF/c-Met pathway to promote cancer progression. Additionally, MMPs have demonstrated potential cancer-inhibiting effects [47, 48]. Our results were not significant, a finding similar to the findings of Paulina Vaitkiene [49]. However, the high expression group of TIMP1 showed a poor prognosis, similar to the findings of Charlotte Aaberg-Jessen [40]. This phenomenon may be due to the insufficient number of patients and requires further work.

TIMP-2 is a unique member of the TIMP family because it directly inhibits endothelial cell proliferation instead of metalloproteinase activity [50]. TIMP2 has recently made adequate progress among biomarkers of renal injury [51, 52]. Regarding cancer, the current findings show that the role of TIMP2 in prognosis depends on the tissue of origin of the tumor [53]. However, epigenetic silencing of TIMP2 can promote invasion of ovarian cancer (OC) [54]. Concerning renal cell carcinoma (RCC), TIMP2 is inversely correlated with markers of tumour progression [55]. However, TIMP2 is a poor prognostic marker in gastric cancer [56], in contrast to its role in colorectal cancer [57]. TIMP2 is involved in glioma inhibition by different drugs as an intermediate link in the upregulated expression in different studies [58, 59]. However, regarding GBM, laboratory evidence has shown that TIMP2 promotes MMP2 activation and GBM cell invasion [60]. This finding indicates that the effect of TIMP2 on GBM is more complex than previously thought in the real environment. We showed that TIMP2 expression was positive, although not significantly, associated with the prognosis of GBM patients (Fig. 3A). This result may also be a manifestation of its complex role.

TIMP3 is the only TIMP family gene with an affinity for proteoglycans in the ECM, and it has the broadest range of substrates [61]. TIMP3 frequently leads to a poor prognosis because of silencing in tumours [46]. In addition to being a marker for predicting cancer progression, TIMP3 can also be used as a therapeutic target for cancer and a marker of tumour sensitivity to drugs [62–64]. In conclusion, TIMP3 has been shown to play a role in inhibiting cancer progression in numerous studies. A similar role has been reported for TIMP3 in glioma. MicroRNA 21 (miR-21) enhances the malignant degree of glioma by inhibiting TIMP3 expression [65]. In another study, upregulated expression of TIMP3 emerged in euxanthone-inhibited GBM cell lines [66]. TIMP3 can suppress angiogenesis by binding to vascular endothelial growth factor receptor 2 (VEGFR2) and disrupting the interaction of VEGF with VEGFR2 and downstream signalling [67]. Additionally, TIMP3 induces apoptosis and inhibits cell proliferation [68]. These functions all underlie TIMP3 as an inhibitor in various cancers, including GBM. Our findings also reflect these characteristics of TIMP3. TIMP3 overexpression was associated with better prognosis in GBM patients for both OS and DSS (Fig. 3A). This finding provides evidence for the positive impact of TIMP3 on the prognosis of GBM patients and validates the potential of TIMP3 as an intermediate link in drug therapy for various malignancies, including GBM, for further development. We also list genes associated with differential expression of TIMP3 in GBM that may develop similar application values to TIMP3 because of its similar role in GBM (Fig. 3D). Existing studies also demonstrated similar potential for these genes. For example, TOP2 is a member of the TOB/BTG protein family and inhibits cell proliferation and stimulates cell differentiation [69]. The dysregulation of TOP2 is associated with the development of hepatocellular carcinoma (HCC) [70]. MTMR3 belongs to the myotubularin-related protein family and is a target gene of various miRNAs that regulate tumour development [71, 72]. MTMR3 modulates the proliferative phenotype and apoptosis of glioma cells by mediating the Wnt/β-catenin pathway and correlates with glioma grade [73]. EIF4ENIF1 encodes a nucleocytoplasmic shuttle protein for the translation initiation factor eIF4E that constitutes a repressor complex involved in neurogenesis and confers resistance to DNA damaging agents through interaction with eIF4 [74–76].

TIMP4 is expressed only in specific tissues of the human body, and this restricted expression implies its functional specificity compared with other family members [77]. Thus, relatively few studies have investigated the role of TIMP4 in tumours, making its role in some tumours unclear in published studies. In studies on renal cell carcinomas (RCCs), TIMP4 showed opposite expression patterns in clear cell and papillary cell carcinomas [78]. regarding breast cancer, in contrast to Wang’s study in which TIMP4 played a role in stimulating tumorigenesis [79], Jiang’s study showed that TIMP4 inhibited tumour growth and invasion [80]. Recent studies have revealed that TIMP4 is involved in the inhibitory effect of various substances on cancer, showing a negative regulatory role in the progression of various tumours. Low TIMP4 expression induces resistance to chemotherapeutic agents in non-small cell lung cancer (NSCLC) [81]. Stable TIMP-4 knockdown promotes the proliferation and migration of well-differentiated liposarcoma (WDLS) [82]. In bladder and prostate tumours, TIMP4 is downregulated by chemokine (C-X-C motif) ligand 1 (CXCL1) as an intermediate link to maintain tumour growth [83]. Regarding the relationship between TIMP4 and glioma progression, the directivity of the available findings is inconsistent. TIMP4 binds CRN2 (an actin filament binding protein) and promotes perivascular invasion of GBM cells [84]. High TIMP4/CD63 coexpression contributes to the poor prognosis of GBM patients [85]. TIMP4 has been shown in Pullen’s study to have an inverse expression relationship with MMP1, which performs enhanced tumorigenic function in GBM [86]. TIMP4 is also downregulated during human herpesvirus 6 (HHV-6) promotion of glioma development and progression [87]. In our study, high TIMP4 expression was associated with better OS in GBM patients. Although the relationship between the expression level of TIMP4 and DSS was not significant, the effect of TIMP4 expression on DSS was positive (Fig. 3A). This result may be explained by the insufficient number of enrolled patients. Notably, a significantly positive relationship was found between TIMP4 and TIMP3 expression (Fig. 3B), warranting further investigation and providing evidence for the effect of TIMP4 on the prognosis of GBM patients. Finally, a list of genes highly correlated with TIMP4 expression is also provided for exploration (Fig. 3D). Recent studies provide a basis for the further exploration of these genes. For example, SCRG1 (Scrapie Responsive Gene 1) is involved in neurodegeneration and autophagy and is associated with transmissible spongiform encephalopathy. It can also maintain the stemness of stem cells through the SCRG1/BST1 axis [88–90]. SHISA4 belongs to the SHISA family and mediates synaptic transmission in the central nervous system by constituting an auxiliary subunit of the AMPA receptor [91]. COMMD2 is the second member of the copper metabolism MURR1 domain protein family, in which COMMD1 inhibits GBM cell proliferation and promotes lung cancer cell apoptosis [92, 93].

Based on the clinical and molecular pathological features of GBM, multiple classification methods exist. Our study demonstrated that the expression of TIMP family genes did not show a correlation with sex. The gene encoding isocitrate dehydrogenase (IDH) distinguishes GBM patients from IDH-mutant (IDH-mt) and wild-type (IDH-wt) GBM patients [94]. Existing studies have shown that the IDH mutant body type has a better prognosis and therapeutic effect than the IDH wild-type, accounting for approximately 10% of GBM [95, 96]. TIMP1 expression was markedly elevated in wild-type GBM, corresponding to patients with high TIMP1 expression showing a worse prognosis, although not significantly. TIMP2 expression did not differ greatly between the two groups, corresponding to the close results of the two prognostic subgroups. The MGMT gene is associated with DNA repair, and its methylation produces gene silencing, allowing glioblastoma patients receiving chemotherapy to achieve a better prognosis [97]. We observed even lower TIMP1 expression in MGMT-methylated patients, a finding that may also be responsible for the better prognosis of patients with downregulated TIMP1 expression. GBM was originally divided into four subtypes according to gene expression profiles—classical, mesenchymal, proneural, and neural [98]. However, some studies have found that the neural subtype is produced because normal samples are contaminated [99]. Mutations of EGFR often occur in the classical subtype, and it and the mesenchymal subtype are more likely to be affected by aggressive treatment [96]. GBM patients with proneural subtypes frequently have rare genetic mutations, particularly IDH mutations with better prognosis [99]. Our study lays the foundation to evaluate the mechanism and how differential expression of TIMPs affects the survival of GBM patients with central subtypes.

The tumour microenvironment (TME) has gained increasing attention because it regulates tumour progression and significantly affects the treatment response [100, 101]. The infiltration of immune cells in the TME promotes or antagonizes tumours [102]. We analysed the effect of TIMP family members on immune cell infiltration in GBM with the TIMER database and found that the expression and CNV of TIMPs are closely related to the infiltration of six immune cells. These four members can be identified in GBM because of differences in multiple microenvironment indicators, including tumour purity. Our results help to further reveal the molecular mechanism of GBM progression and are expected to use this gene family as a link in immunotherapy to specifically affect the GBM tumour microenvironment and improve the treatment of GBM.

Our study has some limitations, primarily because our data were all obtained from public databases, making some heterogeneity in the source and use of the data inevitable. Our results mainly provide a direction and basis for studies targeting TIMPs in GBM. Further experimental and clinical validation for translational purposes is warranted.

## Conclusion

We conducted a systematic study on the expression, prognosis, and immune infiltration of TIMPs in GBM. Our results confirm that the expression of all four members of the TIMP family is upregulated in GBM. High expression of both TIMP3 and TIMP4 can be used as a predictor of better survival in GBM patients and a positive relationship was found between them. These findings led to the identification of related genes for further analysis. Finally, the expression of this gene family was associated with the infiltration of immune cells, including CD4+ T cells, macrophages, neutrophils, B cells, CD8+ T cells, and dendritic cells, to varying degrees. This effect provides new exploration directions for research on the TIMP family and GBM.

## Supplementary Information

**Additional file 1.**

## Data Availability

Publicly available datasets were analyzed in this study. Institutional review board approval was not demanded in our study for all database is publicly available; This data can be found in following website. Oncomine: http://www.oncomine.org; GEPIA2: http://gepia2.cancer-pku.cn/; OSgbm: http://bioinfo.henu.edu.cn/GBM/GBMList.jsp; LinkedOmics: http://www.linkedomics.orglogin.php; STRING: https://string-db.org; GeneMANIA: http://www.genemania.org; Enrichr: https://maayanlab.cloud/Enrichr/; KEGG: https://www.kegg.jp/kegg/kegg1.html; cBioPortal: http://cbioportal.org; TIMER: https://cistrome.org/.

## Abbreviations

TIMPs: The tissue inhibitors of metalloproteinases
ECM: Extracellular matrix
MMP: Matrix metalloproteinases
GBM: Glioblastoma
OS: Overall survival
DSS: Disease special survival
EPA: Erythroid-potentiating activity
NSCs: Neural stem cells
bFGF: Basic fibroblast growth factor
GEPIA: Gene Expression Profiling Interactive Analysis
TCGA: The Cancer Genome Atlas
GTEx: Genotype-Tissue Expression
GEO: Gene Expression Omnibus
CGGA: Chinese Glioma Genome Atlas
KM: Kaplan-Meier
HR: Hazard ratio
IDH: Isocitrate dehydrogenase
PPI: Protein-protein interactions
KEGG: Kyoto Encyclopedia of Genes and Genomes
CNV: Copy number variation
RCC: Renal cell carcinoma
miR-21: MicroRNA 21
VEGFR2: Vascular endothelial growth factor receptor 2
RCC: Renal cell carcinomas
NSCLC: Non-small cell lung cancer
WDLS: Well-differentiated liposarcoma
HHV-6: Human herpesvirus 6
